# Application of Xylo-Oligosaccharide-Rich Gel Emulsion as a Fat Replacer in Sausages

**DOI:** 10.3390/foods13223625

**Published:** 2024-11-14

**Authors:** João L. F. Paschoa, Patrícia F. Ávila, Gilmar F. da Costa, Ana Paula B. Ribeiro, Renato Grimaldi, Rosiane L. da Cunha, Marise A. R. Pollonio, Rosana Goldbeck

**Affiliations:** 1 Laboratory of Bioprocess and Metabolic Engineering, School of Food Engineering, University of Campinas (UNICAMP), Campinas 13083-862, SP, Brazil; j158032@dac.unicamp.br (J.L.F.P.);; 2 Meat and Process Laboratory, School of Food Engineering, University of Campinas (UNICAMP), Campinas 13083-862, SP, Brazil; g264686@dac.unicamp.br (G.F.d.C.); pollonio@unicamp.br (M.A.R.P.); 3 Oils and Fats Laboratory, School of Food Engineering, University of Campinas (UNICAMP), Campinas 13083-862, SP, Brazil; anabadan@unicamp.br (A.P.B.R.); grimaldi@unicamp.br (R.G.); 4 Process Engineering Laboratory, School of Food Engineering, University of Campinas (UNICAMP), Campinas 13083-862, SP, Brazil

**Keywords:** xylo-oligosaccharides, gel emulsions, pork fat replacement, prebiotics, functional products

## Abstract

Xylo-oligosaccharides (XOS) are functional oligosaccharides obtained from xylan present in lignocellulosic material. This study investigated the effects of replacing pork fat with functional xylo-oligosaccharide gel emulsion (XGE) on the chemical and physical structure of developed meat products. The product’s centesimal composition, energy value, pH, color parameters, and microstructure were analyzed. The results showed that replacing pork fat with XGE reduced the total lipid content by approximately 30%, and provided a desirable lipidic profile with reduced thrombogenicity and atherogenicity indices. A microstructure analysis showed that products with partial and full pork fat replacement presented a more compact structure than the control formulation. Thus, XGE is a viable alternative to replace pork fat in meat products since it maintains similar physicochemical and technological properties to the original products and contributes to the development of healthier meat products with prebiotic properties, lower fat content, and, consequently, lower energetic value.

## 1. Introduction

Meat and meat products contain high-quality proteins and several micronutrients (e.g., iron, zinc, selenium, and B-complex vitamins) [1]. The addition of saturated fat during the production of meat emulsions positively influences the quality of these products, contributing to several technological and sensory properties (e.g., emulsion stability, texture profile, reduction of water loss during cooking, and flavor) [2]. Despite these technological and sensory benefits, fat-saturated products cause undesirable health effects such as obesity, hypertension, and cardiovascular diseases. These chronic illnesses have driven changes or reductions in lipid profile through partial or total replacement of saturated fats in products of animal origin, especially meat products [3,4].

Vegetable oils have emerged as the primary alternative to saturated fat in meat products, as they are sources of monounsaturated, polyunsaturated, and omega-3 fatty acids [5]. However, vegetable oils have a low melting point, and their direct addition can negatively impact the physicochemical, technological, and sensory properties of the products to which they are added. In this context, technological strategies for structuring these vegetable oils can make them capable of mimicking the properties of saturated fat [6,7]. Vegetable oils structured using emulsion gels have been widely reported in studies in recent years as a replacement for saturated fat in meat products [8,9,10,11,12,13,14]. These studies demonstrate that emulsion gels can improve the stability, texture, and water-holding capacity of meat products, in addition to contributing to a healthier formulation. The application of this technology also facilitates the incorporation of unsaturated fatty acids, such as omega-3, promoting health benefits.

Fat replacement in meat products has been an area of extensive research for over three decades, resulting in significant advancements in understanding how to improve the nutritional quality and functional properties of these products. Recent studies have shown that substituting animal fat with plant-based oils and functional ingredients, such as starch-based gels and proteins, can enhance the emulsion stability and water-holding capacity (WHC) while reducing the saturated fat content [3,5,6]. Additionally, the incorporation of bioactive compounds, such as XOS, not only improves the lipid profile but also contributes to the health benefits of meat products by promoting gut health. Furthermore, consumer demand for healthier and functional food options has accelerated the development of reformulated meat products that maintain sensory attributes comparable to traditional formulations [7,11,14].

Lipid profile reformulation in meat products intends to make them healthier. Vegetable oils have thus emerged as the main alternative to replace saturated fats since they are a source of monounsaturated and polyunsaturated fatty acids; however, this strategy can compromise the quality of such products regarding their technological and sensory properties [8,9]. Hence, new possibilities have been evaluated to enhance its viability which included mainly structuring these oils into gel forms or gel emulsions, as they present similar properties to saturated fat. Moreover, the use of structuring compounds such as proteins and polysaccharides can contribute to developing meat products with improved functional properties [4,5].

A structured emulsion is characterized by a gel-like network structure in the continuous phase, which confers greater stability by entrapping the dispersed phase. This approach has great potential for applications in the search for healthier options [15,16]. The need for a healthier lifestyle has sparked great interest in the use of prebiotic compounds to replace fat and sugar in various food preparations (e.g., meat, baked goods, dairy products, and beverages) [17].

Sausage processing involves grinding and mixing the meat with fat and seasonings, stuffing it into casings, and cooking or curing it to stabilize it. Techniques vary for fresh, cooked, or fermented types, aiming for specific texture and flavor. Condiments such as salt and nitrite are commonly used for protein extraction, preservation, and color [18].

Myofibrillar proteins in meat, especially actin and myosin, are essential for emulsified meat products, as they play a crucial role in stabilizing the matrix formed by fat and water due to their emulsifying capacity, which ensures cohesion and texture in the final product [1]. Added animal fat, in addition to contributing to flavor, tenderness, and mouthfeel, is traditionally used to provide the desirable texture in meat products [1,2]. However, the partial or total replacement of animal fat with gels rich in functional compounds, such as xylo-oligosaccharides (XOS), can significantly improve the lipid profile of the product, reducing the saturated fat content and adding prebiotic benefits that promote intestinal health. Thus, the development of meat emulsions that integrate these functional compounds not only contributes to a healthier product, but also increases added value by meeting growing consumer demands for foods with functional properties [5,6,7].

Xylo-oligosaccharides (XOS) are functional oligosaccharides obtained from xylan present in lignocellulosic material and are reported to be potential prebiotic ingredients [19]. Although little explored in the meat technology field, XOS could be used in food gel emulsions for saturated fat replacement in meat product formulations due to their excellent technological properties (e.g., high water holding capacity, wide pH range, and temperature stability) [20,21]. Additionally, XOS offer numerous health benefits due to their selective metabolism promoted by Bifidobacteria, resulting in increased production of volatile fatty acids and anti-gastric ulcer activity [22,23].

Given the above, this work evaluated the effects of partial and total pork fat replacement with functional xylo-oligosaccharide gel emulsion (XGE) on the physicochemical, technological, lipidic profile, and microstructural properties of emulsified meat products (sausage).

## 2. Materials and Methods

### 2.1. Raw Material

Pork (*M. longissimus thoracis*) meat (72.05% moisture, 3.42% lipids, 18.3% protein, and 0.88% ash) and pork fat (5.23% moisture, 88.58% lipids, 5.94% protein, and 0.25% ash) were obtained from a local market in Campinas city, Brazil. Functional xylo-oligosaccharide gel emulsion (XGE) (50% sunflower oil, 0.4% isolated soy protein, 4.85% carrageenan, 44.75% XOS-rich hydrolysate—57 g·L^−1^; pH 3.5) was used in this study. XOS-rich hydrolysate was produced previously by enzymatic hydrolysis of xylan extracted from sugarcane bagasse, according to Paschoa et al. [24].

Soy protein isolate (pH 6.2–7, at least 90% protein, Supro^®^ 596 P, Kerry do Brasil, Campinas, Brazil) and carrageenan (pH 8–10, GENUGEL^®^ MB-530 F, CP Kelco, Limeira, Brazil) were donated by the respective companies. Sunflower oil (Concordia, ADM^®^) was obtained from a local market in Campinas, Brazil.

### 2.2. Preparation of Xylo-Oligosaccharides Gel Emulsion (XGE)

XGE was prepared using soy protein isolate (SPI), carrageenan (CAR), hydrolysate containing xylo-oligosaccharides (XOS), and sunflower oil. For preparation, the addition of sunflower oil was fixed at 50% (*w*/*w*) and the remaining 50% (*w*/*w*) represented the aqueous solution containing the other ingredients. The mixture of soy protein isolate (SPI), hydrolysate (XOS), and distilled water was previously heated and homogenized (60 °C, 5 min, 500 rpm) under magnetic stirring and then cooled to 25 °C using an ice bath. In a 50 mL Falcon, the homogenized and cooled SPI-XOS mixture was added with the aid of a bench homogenizer (TURRAX) (10,000 rpm, 2 min). Sunflower oil was gradually added with a Pasteur pipette until fully incorporated into the mixture, forming an emulsion. Carrageenan (CAR) was then added to the emulsion using the same homogenizer (10,000 rpm, 1 min) until reaching a gel-like texture.

### 2.3. Model System Elaboration

The pork meat and fat were previously ground in a meat grinder. The experiment was conducted with three different formulations (Table 1).

The control formulation (FC) consisted of 100% pork fat, while the other formulations were prepared by replacing 50% (F1) and 100% (F2) of the pork fat with the emulsion gels. Ground pork, salt, and part of the ice were homogenized in a food processor for 10 s, followed by the addition of the additives (sodium tripolyphosphate, sodium nitrite, and sodium erythorbate) and homogenized for 30 s. The mixture was then emulsified with the pork fat or gel emulsions and the remaining ice. Emulsification continued for another 30 s. The batter temperature did not exceed 12 °C in any of the formulations. Forty grams of each formulation were weighed into 50 mL Falcon tubes and centrifuged at 3600 rpm for 2 min. The samples were boiled in a water bath until the internal temperature reached 75 °C and then immediately cooled in an ice bath and stored in a refrigerator at 4 °C. All analyses were conducted within 24 h of preparation.

### 2.4. Analysis of Model Systems

#### 2.4.1. Chemical Composition

Moisture, protein, and ash contents were analyzed in triplicate according to AOAC [25]. The fat content was assessed in triplicate according to Bligh and Dyer [26]. The carbohydrate content was estimated by calculating the difference between the other components. The energy content was calculated based on 9 kcal/g for fat and 4 kcal/g for protein and carbohydrate [27].

#### 2.4.2. Emulsion Stability

The emulsion stability of the different meat model systems was estimated in triplicate per sample according to Hugles, Cofrades, and Troy [28] with some modifications. A total of 20 g of the meat mass was weighed into Falcon tubes and centrifuged for 2 min at 3600 rpm. The samples were then cooked in a warm water bath until the internal temperature reached 75 °C. Subsequently, the samples were cooled on ice and the liquid released was weighed into 50 mL beakers and dried at 105 °C for 24 h. The percentage emulsion stability (ES %), fat released (FAT%), and percentage of liquid released (TEF %) were calculated as described in Equations (1)–(3):

(1) $$\mathrm{ES}\;(\%)=100-\frac{\left(\mathrm{Weight}\;\mathrm{of}\;\mathrm{centrifuge}\;\mathrm{tube}\;\mathrm{and}\;\mathrm{sample}\right)-\left(\mathrm{Weight}\;\mathrm{os}\;\mathrm{centrifuge}\;\mathrm{tube}\;\mathrm{and}\;\mathrm{pallet}\right)}{100\times \mathrm{Sample}\;\mathrm{weight}\;\left(g\right)}$$

(2) $$\mathrm{FAT}\;\%=\frac{\mathrm{Weight}\;\mathrm{of}\;\mathrm{crucible}\;+\;\mathrm{dried}\;\mathrm{supernatant}\;\left(g\right)}{\mathrm{ES}\;\left(g\right)}\times 100$$

(3) $$\mathrm{TEF}\;\%=\frac{\mathrm{ES}\;}{\mathrm{Sample}\;\mathrm{weight}\;\left(g\right)}\times 100$$

#### 2.4.3. pH, Water Activity (a_w_) and Color Determination

The pH was measured using a HI 99,161 pH meter (HANNA Instruments, Woonsocket, RI, USA). Three readings of each sample were taken. Water activity (a_w_) was measured using an Aqualab water activity meter (Decagon, Pullman, USA). Color parameters were determined using a MiniScan Xe Plus spectrophotometer model MSXET (Hunter-Lab, Reston, VA, USA) based on the CIELAB color system. The model system was sliced into 0.5 cm thickness. Readings were taken in triplicate, with the model system sliced into 0.5 cm thickness at 25 °C for three replicates. The whiteness indicator (*WI*) was calculated according to the equation proposed by Lohman and Hartel [29] (Equation (4)).

(4) $$WI=100-\sqrt[{2}]{{\left(100-L\right)}^{2}+{\left(a\right)}^{2}+{\left(b\right)}^{2}}$$

#### 2.4.4. Pressed Juice

Pressed juice was determined following the methodology described by Lucherk et al. [30]. Sausage samples (1 cm cubes) were compressed using a TA-XT2i texturometer (cylindrical probe 2 cm diameter) (Texture Technologies Corp., Scarsdale, NY, USA). The percentage of fluid weight lost during compression was calculated as the pressed juice percentage using the following equation:

(5) $$\%\;Press\;juice=\frac{Wf-Wi}{Ws}\times 100$$

where *Wf* is the final weight of the filter paper, *Wi* is the initial weight of the filter paper, and *Ws* is the weight of the samples.

#### 2.4.5. Texture Profile Analysis (TPA)

A TPA analysis was conducted at room temperature (22–25 °C) using a TA-xT2i texture analyzer (Texture Technologies Corp., Scarsdale, NY, USA), according to Horita et al. [31]. Twelve cylinders obtained from the samples with 20 mm height and 22 mm diameter were subjected to two consecutive uniaxial compression cycles at 50% of their initial height at a constant velocity of 1 mm/s, using a P-35 probe (35 mm in diameter, stainless steel). The parameters evaluated consisted of hardness (N), springiness (mm), cohesiveness (dimensionless), and chewiness (N⋅mm).

#### 2.4.6. Scanning Electron Microscopy

The sausage microstructure evaluation was conducted using a high vacuum scanning electron microscope TM 4000 Tabletop Microscope, with a magnitude of 15× to 30,000× and a 15 kV acceleration voltage (Hitachi High Technologies, Japan), using a non-destructive technique dispensing sample preparation at magnifications of 200× and 500× (digital zoom: 2× and 4×). The product was cut into a standard size (2 cm × 2 cm) with a 2 mm thickness, placed in a stub, and analyzed in the modular equipment at 5 kV and 15 kV.

#### 2.4.7. Fatty Acid Composition (FAC) and Nutritional Parameters

The FAC was calculated using a GC equipped with a capillary column (CGC 68,650 Series GC System, Agilent, Santa Clara, CA, USA) and a flame ionization detector. Methyl esters in the fatty acids were separated using an Agilent DB-23 capillary column (50% cyanopropyl-methylpolysiloxane), dimensions of 60 m, Ø int 0.25 mm, and 0.25 μm film thickness following the chromatographic conditions reported by Paglarini et al. [32].

The nutritional characteristics of the sausages were evaluated by determining the PUFA and SFA ratios, as well as the indexes of atherogenicity (AI) and thrombogenicity (TI). AI was calculated using the following equation: (C12:0 + 4 × C14:0 + C16:0)/(C16:1 + C17:1 + C18:1n − 9 + C20:1 + C18:2n − 6 + C18:3n − 3) and TI (C14:0 + C16:0 + C18:0)/[(0.5 × MUFA) + (0.5 × Σ n − 6) + (3 × Σ n − 3) + (Σ n − 3/Σ n − 6)] [33].

#### 2.4.8. Statistical Analyses

Results underwent analysis of variance (ANOVA) at a 95% confidence level (*p* < 0.05), considering formulations as a fixed effect and replicates as a random effect. Significant differences between formulations were analyzed by Tukey’s test at a 5% significance level, using Minitab^®^ 21.1.0 software for data analysis.

## 3. Results and Discussion

### 3.1. pH and Color Batter Determination

Batter pH values varied significantly (*p* < 0.05) between formulations (Table 2). F2 formulation, which replaced 100% of the pork fat with the same amount of XGE, presented the lowest pH value. Color parameters showed no statistically significant difference (*p* < 0.05) between formulations for lightness (L*); however, XGE addition affected the other color parameters related to chromaticity (a* and b* values).

**Figure 1:**
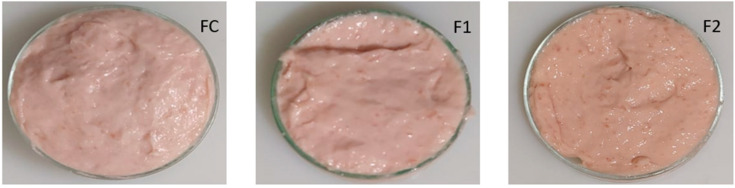
Fresh batter formulations with xylo-oligosaccharide gel emulsion (XGE). Formulation control—FC = 100% pork fat. F1 = 50% pork fat + 50% XGE. F2 = 100% XGE.

Values of (a*) and (b*) differed only between FC and F2, indicating that XGE at higher concentrations produces a slight change in the color of the formulation. Conversely, the whiteness indicator (W) decreased significantly between FC and F2 but not between FC and F1 (*p* < 0.05). Figure 1 illustrates the batters obtained for each formulation.

These significant variations between formulations did not affect the quality of the product, particularly the color parameters (between F1 and FC). Felisberto et al. [34] reported similar results when evaluating the effect of probiotic ingredients on the physicochemical properties of low-sodium, low-fat meat emulsions. Additionally, the gel emulsions remained stable (no visual phase separation).

### 3.2. Model System Analysis

#### 3.2.1. Centesimal Composition and Energy Value

Table 3 summarizes the centesimal composition and energy value of the meat product (model system) produced. Systems with partial (F1) and total (F2) pork fat reduction presented higher moisture content than the control formulation (FC), probably due to the replacement of pork fat with XGE (higher moisture) in these formulations. Similar results were found by Pagliarini et al. [35], with a 21% fat reduction using inulin emulsion gel compared with the control formulation, and dos Santos et al. [36], with a 30% fat replacement using bamboo fiber- and inulin-containing emulsion gels compared with the control.

The products with partial (F1) and total (F2) pork fat replacement with XGE showed a significant lipid content reduction compared with the control formulation (FC). F2 presented a nearly 30% reduction, characterizing this meat product formulation as having a low total fat content [37,38]. Fan, Zhou, and Cao [39] reported similar results when investigating the use of β-glucan collagen peptide gels as a substitute for pork fat in sausages.

Protein values in F1 and F2 were significantly lower compared with the control (FC), a result possibly explained by the increased moisture content of these formulations and the low protein content (around 0.4% *w*/*w*) of XGE. Nonetheless, all formulations presented a protein content above the 12% *w*/*w* minimum established by Brazilian legislation [37] for emulsified meat products.

Replacing pork fat with a gel emulsion containing XOS resulted in a reduction in the lipid and protein content, which offers opportunities to optimize the characteristics of the final product. The reduction in lipids can make the nutritional profile lighter and more appealing to health-conscious consumers, while the functional action of XOS can compensate for the loss of protein, helping to stabilize the emulsion and adding value to the product with its functional properties [4,14,15].

**Figure 2:**
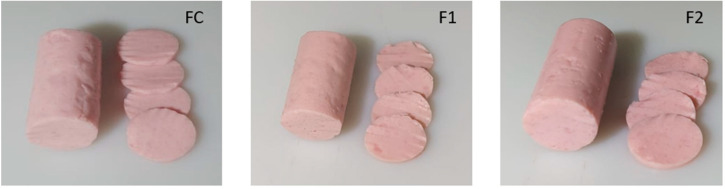
Model system after cooking with xylo-oligosaccharide gel emulsion (XGE). Formulation control—FC = 100% pork fat. F1 = 50% pork fat + 50% XGE. F2 = 100% XGE.

F1 and F2 showed significantly higher total ash and carbohydrate contents than the control formulation (FC). F2 had the highest ash concentrations, which may be associated with the XOS content in the gel. XOS are resistant to acidic pH and are difficult to degrade during food processing involving heat and even during digestion [22,40,41]. XGE contained carrageenan, a linear polysaccharide used as a gelling agent in gel emulsions [35], at a concentration of 4.85%, which contributed to increases in the carbohydrate content in the pork fat-replaced formulations. Due to total pork fat replacement by XGE, F2 had the lowest energy value, a total reduction of 23% when compared with the control (FC). Figure 2 depicts the model systems after cooking, in both whole and sliced form. The absence of visual differences in both formulations suggests that the partial (F1) and total (F2) fat substitution with XGE did not interfere with this aspect.

#### 3.2.2. Physicochemical Parameters, Emulsion Stability, and Texture Profile

Table 4 presents the characterization of the physicochemical and technological properties of the model systems produced. pH values differed statistically for all formulations, ranging from 5.97 to 6.18. Partial (F1) and total (F2) pork fat replacement showed lower pH values than the control (FC) due to the more acidic characteristics of XGE. Kin et al. [2] and Martins et al. [42] report that the acidity of gel emulsion ingredients can interfere with the pH values of meat emulsions when used as a pork fat replacement. Water activity (aw) remained unaffected by the formulations, suggesting that XGE did not increase the bound water content in the meat emulsions.

Pressed juice is a key parameter for assessing the quality of low-fat meat products and is measured by the amount of water released during the compression of the sample, which is strongly associated with the product’s stability. The pressed juice values did not differ between formulations F1 and F2 even though F2 had higher moisture content than F1, but both differed significantly from the control formulation (FC), indicating that XGE did not favor water retention. However, since F1 and F2 presented higher moisture content values, more water was released from the meat matrix during compression (Table 3).

Both formulations presented higher emulsion stability compared with the control (FC). Replacing the pork fat with XGE may have increased meat emulsion stability, but the greater juice release observed could contribute to the sensory perception of the product.

All color parameters (L*, a*, b*) were affected by the replacement of pork fat with XGE. L* values increased, with F2 differing significantly from FC. The gel emulsion droplet diameters are smaller when compared with those of animal fat, generating greater light reflection and consequently increasing the L* values [43]. Redness values (a*) differed between formulations *(p* < 0.05), with a* values reducing as the XEG content increased. This may be associated with the reduced lipid content between the samples [44]. Carvalho et al. [45] reported similar results when observing an increase in a* values following fat replacement with curcumin in the gel emulsion formulation of sausages. b* values showed a significant increase between the formulations (FC, F1, and F2). Despite these differences, whiteness values showed no significant differences between samples (*p* < 0.05).

Meat emulsion stability (ES) is a strong indicator of a successful reformulation, as it is directly associated with the production process [36]. Adding XGE to the reformulation of the model system significantly increased stability (Table 4). F1 and F2 presented no ES (%) differences between them (*p* < 0.05); however, their values were superior to the control formulation (FC). Carrageenan, a hydrocolloid extracted from seaweed, is an important component in XGE formulation. This polysaccharide is widely used in the food industry as a stabilizer and gelling agent, increasing the model system’s water-holding capacity. An improved stability of the formulations with pork fat replacement was observed after heat treatment (process condition) [46].

Tripolyphosphate Sodium (TPS) is widely used in processed meat products due to its ability to enhance emulsion stability and reduce cooking loss. This functionality is directly linked to its impact on water retention and decreased fat release during heating. TPS increases protein solubility, which, when combined with changes in pH and ionic strength within the matrix, strengthens the protein gel structure. This reinforced structure is essential for preserving the texture and juiciness of the meat products while minimizing exudate, ultimately contributing to the overall quality of the final product [32,35]. Although some samples showed reduced cooking loss or lower fat exudation, indicating better physicochemical properties, these results are limited due to the use of tripolyphosphate sodium (TPS). This additive is known for enhancing emulsion stability and reducing exudates, which could mask the effects of other ingredients in the formulation.

Low values regarding fat separation from the exuded liquid between formulations indicate that the system was able to trap almost all the lipid phase (pork fat). F1 and F2 showed no differences regarding water separation, but both were lower than FC, thus corroborating the greater stability of the reformulated systems. Despite the higher moisture content, XGE addition increased emulsion stability, consequently reducing water exudation after cooking.

As for the texture profile (Table 4), replacing 50% (F1) and 100% (F2) of pork fat with XGE significantly reduced the system hardness compared with the control (FC). Similar results were reported by Pintado et al. [10]. Chewability is the energy required to deform the sample for swallowing and is directly related to hardness. F1 and F2 formulations had significantly lower chewability values (*p* < 0.05) than FC. No significant difference (*p* > 0.05) was observed between the formulations for springiness (Table 4). The texture profile of a meat product is an important property in terms of consumer acceptance, and despite small differences, XGE-containing formulations had a similar texture to the control (pork fat), demonstrating potential as an ingredient for pork fat replacement.

#### 3.2.3. Microstructure

**Figure 3:**
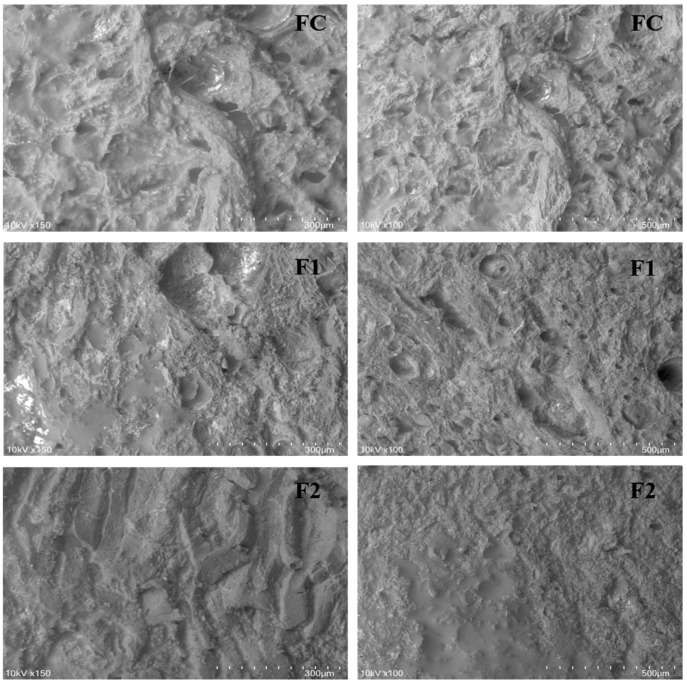
Scanning electron microscopy of the model systems produced. Formulation control—FC = 100% pork fat. F1 = 50% pork fat + 50% XGE. F2 = 100% XGE. Figures are shown in duplicate for each test. XGE—Xylo-oligosaccharides gel emulsion. Figures on the left are at a 300 µm scale and those on the right are at a 500 µm scale.

To assess the morphology of each formulation’s matrix structure, we conducted a scanning electron microscopy (SEM) test (Figure 3).

The formulations with XGE (F1 and F2) showed a progressively denser and more compact topographic structure compared to the control (FC), with the compaction increasing as the fat content was replaced by the XOS-rich gel emulsion. In particular, the 100% XGE formulation (F2) presented a substantial reduction in porosity compared to F1, which contained 50% XGE. This reduction in porosity suggests that the XOS in the gel emulsion significantly enhances the water-binding capacity of the network. By integrating into the protein network, XOS forms hydrogen bonds with water molecules, effectively trapping moisture and creating a more cohesive and stable structure [35,43].

Additionally, the increase in compaction in the XGE formulations can contribute to improved textural attributes, as the gel network supports a firmer, more stable structure that can withstand mechanical stresses during processing and storage. This enhanced structural integrity not only provides a potential advantage in maintaining the shape and consistency of low-fat products but also offers an appealing mouthfeel, which is often challenging to achieve in reduced-fat formulations. By improving hydration and structural stability, XGE serves as an effective fat replacer, maintaining similar functional and sensory properties to traditional fat-based formulations, while also adding the health benefits of the prebiotic XOS [21,22,34,40].

#### 3.2.4. Fat Acids Profile and Nutritional Parameters

Table 5 compares the fatty acid composition of four samples: FC (100% animal fat), F1 (50% replacement of animal fat with an emulsified gel rich in XOS and sunflower oil), F2 (total replacement of animal fat with an emulsified gel rich in XOS and sunflower oil), and sunflower oil. This analysis investigates how replacing animal fat with gel emulsions influences a product’s fatty acid profile and health indices. FC contained the highest amount of SFAs (32.22%), followed by F1 (26.21%) and F2 (18.22%), while sunflower oil presented the lowest SFA content (12.06%). Reducing SFAs is beneficial for one’s health, since high levels are associated with an increased risk of cardiovascular disease [47]. FC had the highest amount of MUFAs (50.47%), with F1 (39.35%) and F2 (36.85%) showing a significant reduction. Sunflower oil contained 33.64% MUFAs. MUFAs are beneficial for cardiovascular health as they help to reduce LDL cholesterol and increase HDL cholesterol [48].

The percentage of PUFAs increases significantly with animal fat replacement. FC presented 15.83% PUFAs, followed by F1 (33.46%) and F2 (44.89%), with sunflower oil showing the highest value (53.53%). PUFAs, especially omega-3 and omega-6, are essential for cardiovascular health and brain function [49]. FC had a PUFA/SFA ratio of 0.49, which increased to 1.28 in F1 and 2.46 in F2, and presented higher values in sunflower oil (4.44). A higher ratio is preferable and is associated with a lower risk of coronary heart disease [50].

FC showed the highest AI (0.51) and TI (0.74) values, indicating greater atherogenic and thrombogenic risk. These indices decrease with partial and total animal fat replacement: F1 (AI: 0.48, TI: 0.50) and F2 (AI: 0.34, TI: 0.31). Sunflower oil had the lowest values (AI: 0.20, TI: 0.16). Reducing the IA and TI values is essential to reducing the risk of cardiovascular disease [33,51].

Recent studies indicate that replacing animal fat with gel emulsions containing sunflower oil can significantly improve food products’ fatty acid profile, increasing PUFA percentage and reducing SFAs, AI, and TI values. These changes not only improve cardiovascular health, but also meet the growing demand for healthier and more sustainable products without significantly compromising their sensory and technological characteristics [52,53].

Replacing animal fat with gel emulsions containing sunflower oil significantly improves the fatty acid profile, increasing unsaturated fatty acids and reducing saturated fatty acids and negative health indices. This promising approach to producing healthier food products contributes to reducing the risk of chronic diseases without sacrificing the sensory quality of the food [10,53]. In addition to reducing fat, the use of hydrogels is a good strategy for increasing the bioaccessibility of bioactive compounds [15] such as XOS, which increases healthiness and enables the development of new functional products.

## 4. Conclusions

The replacement of pork fat with XOS-rich gels resulted in emulsified meat model systems with properties similar to those of the control formulation, such as emulsion stability, water activity, juiciness, and texture profile. Furthermore, the addition of XOS-rich gels as a replacement for pork fat may promote a healthier claim due to the new fatty acid profile obtained. The use of XOS-rich gels in meat products is still poorly explored, and this study demonstrated the potential of these systems to replace pork fat with minimal changes in the physicochemical and technological properties of the emulsified meat model systems. However, future studies should be carried out to explore the potential of these XOS-rich gels as a promising vehicle for providing healthier meat products with functional and nutritional properties, including stability against lipid oxidation, with a main focus on evaluating the sensory properties of the added meat products.

## Figures and Tables

**Table 1:** Formulation of model systems containing 100% pork fat (formulation control—FC), replacement of 50% (F1) and 100% (F2) pork fat by xylo-oligosaccharide gel emulsion (XGE).

Ingredients (%)	Formulations		
	FC	F1	F2
Pork Meat	63	63	63
Pork Fat	20	10	-
XGE	0	10	20
Ice	15.185	15.185	15.185
Salt (NaCl)	1.5	1.5	1.5
TPS	0.25	0.25	0.25
Sodium Nitrite	0.015	0.015	0.015
Sodium erythorbate	0.05	0.05	0.05

TPS: Trypolyphosphate Sodium.

**Table 2:** Effects of pork fat replacement by xylo-oligosaccharide gel emulsion (XGE) on the color and pH parameters of the model system batter.

Formulation	pH	Color Parameters			
		L*	a*	b*	Whiteness
FC	6.07 ± 0.06 ^a^	71.10 ± 0.37 ^a^	3.39 ± 0.16 ^b^	16.13 ± 0.14 ^a^	66.73 ± 0.37 ^a^
F1	5.99 ± 0.03 ^b^	71.31 ± 1.37 ^a^	3.82 ± 0.19 ^ab^	17.25 ± 0.37 ^b^	66.25 ± 1.28 ^ab^
F2	5.78 ± 0.02 ^c^	71.04 ± 0.28 ^a^	3.96 ± 0.46 ^a^	18.09 ± 0.27 ^c^	65.62 ± 0.21 ^b^
**XGE** *	4.58	46.56	−0.03	6.15	46.21

^a–c^: Different letters in the same column indicate significant differences (*p* < 0.05). Data were expressed as mean ± standard deviation. * XGE containing the optimized and validated condition used in the formulation.

**Table 3:** Effects of pork fat replacement by xylo-oligosaccharide gel emulsion (XGE) on the centesimal composition and energy value of the model systems produced.

Centesimal Composition (%)	FC	F1	F2
Moisture	65.97 ± 0.40 ^c^	68.47 ± 0.29 ^b^	71.08 ± 0.16 ^a^
Proteins	17.35 ± 0.06 ^a^	15.66 ± 0.07 ^b^	14.80 ± 0.22 ^c^
Lipids	14.10 ± 0.12 ^a^	12.49 ± 0.08 ^b^	10.11 ± 0.07 ^c^
Ashes	2.45 ± 0.03 ^c^	2.53 ± 0.01 ^b^	2.71 ± 0.03 ^a^
Carbohydrates	0.13 ± 0.05 ^c^	0.85 ± 0.07 ^b^	1.30 ± 0.12 ^a^
Energy value (kcal/100 g)	196.56 ± 1.66 ^a^	176.75 ± 1.14 ^b^	152.79 ± 1.75 ^c^

^a–c^: Different letters in the same row indicate a significant difference (*p* < 0.05). Data were expressed as mean ± standard deviation.

**Table 4:** Effect of replacing pork fat with XOS-rich gel on the color parameters, pH, water activity (a_w_), emulsion stability, and texture profile of the model systems.

Parameters	Formulations		
	FC	F1	F2
pH	6.18 ± 0.03 ^a^	6.12 ± 0.01 ^b^	5.97 ± 0.01 ^c^
a_w_	0.9815 ± 0.001 ^a^	0.9821 ± 0.001 ^a^	0.9816 ± 0.001 ^a^
Pressed Juice (%)	11.80 ± 0.66 ^b^	12.06 ± 0.57 ^a^	13.05 ± 0.59 ^a^
*Color Parameters*			
L*	68.43 ± 0.93 ^b^	69.06 ± 0.50 ^ab^	69.73 ± 0.21 ^a^
a*	7.71 ± 0.28 ^a^	7.33 ± 0.17 ^b^	6.88 ± 0.15 ^c^
b*	10.44 ± 0.30 ^c^	11.34 ± 0.12 ^b^	12.26 ± 0.13 ^a^
W	65.86 ± 0.99 ^a^	66.24 ± 0.51 ^a^	66.62 ± 0.17 ^a^
*Emulsion Stability*			
ES (%)	98.99 ± 0.53 ^b^	99.61 ± 0.25 ^a^	99.72 ± 0.14 ^a^
TEF released (%)	3.44 ± 1.10 ^a^	1.39 ± 0.42 ^b^	1.01 ± 0.54 ^b^
FAT released (%)	0.02 ± 0.01 ^a^	0.01 ± 0.01 ^a^	0.02 ± 0.01 ^a^
*Texture Parameters*			
Hardness(N)	46.86 ± 2.83 ^a^	41.11 ± 2.27 ^b^	43.26 ± 1.95 ^b^
Springiness (mm)	0.89 ± 0.01 ^a^	0.88 ± 0.01 ^a^	0.87 ± 0.01 ^a^
Cohesiveness	0.69 ± 0.02 ^a^	0.68 ± 0.02 ^a^	0.66 ± 0.02 ^a^
Chewiness (N.mm)	28.56 ± 2.42 ^a^	24.63 ± 1.69 ^b^	25.15 ± 1.69 ^b^

^a–c^: Different letters in the same row indicate a significant difference (*p* < 0.05). Data were expressed as mean ± standard deviation.

**Table 5:** Fatty acid composition of FC (Control), F1, and F2 formulations, and the sunflower oil (SFO) sample used in the xylo-oligosaccharide gel emulsion (XGE) formulation.

Fatty Acids	FC	F1	F2	SFO
C10:0 capric	0.08	0.00	0.00	0.00
C12:0 lauric	0.22	0.13	0.41	0.07
C14:0 myristic	1.28	0.79	0.54	0.11
C15:0 pentadecanoic	0.07	0.00	0.00	0.00
C16:0 palmitic	20.63	16.01	10.39	6.44
C17:0 margaric	0.41	0.21	0.00	0.04
C18:0 stearic	9.38	8.50	5.95	4.32
C20:0 arachidic	0.15	0.27	0.30	0.29
C22:0 behenic	0.00	0.30	0.63	0.79
Σ SFA	32.22	26.21	18.22	12.06
C16:1 palmitolenic	2.80	1.11	1.32	0.24
C17:1 margaroleic	0.41	0.14	0.00	0.02
C18:1 oleic	46.31	37.27	35.53	33.26
C18:1 t-oleic	0.21	0.33	0.00	0.00
C20:1 eicosenoic	0.74	0.50	0.00	0.06
C22:1 docosaenoic	0.00	0.00	0.00	0.06
Σ MUFA	50.47	39.35	36.85	33.64
C18:2 linoleic (n − 6)	13.23	31.15	41.49	51.86
C18:2 t-linoleic (n − 6)	0.67	0.62	1.62	0.94
C18:3 linolenic (n − 3)	0.82	1.01	0.61	0.28
C18:3 t-linolenic (n − 6)	0.55	0.31	0.78	0.45
C20:4 lignoceric (n − 6)	0.56	0.37	0.39	0.00
Σ PUFA	15.83	33.46	44.89	53.53
PUFA/SFA	0.49	1.28	2.46	4.44
Thrombogenic index (TI)	0.74	0.50	0.31	0.16
Atherogenic index (AI)	0.51	0.48	0.34	0.20

## Data Availability

The original contributions presented in the study are included in the article, further inquiries can be directed to the corresponding author.
